# Atypical Pelvic Tumors in Children

**DOI:** 10.3390/cancers17040619

**Published:** 2025-02-12

**Authors:** Paulina Sobieraj, Monika Bekiesińska-Figatowska

**Affiliations:** Department of Diagnostic Imaging, Institute of Mother and Child, 01-211 Warsaw, Poland; zaklad.rtg@imid.med.pl

**Keywords:** atypical tumors, pelvis, children, ovarian small cell carcinoma, ovarian Ewing sarcoma, ovarian lymphoma, ovarian angiosarcoma, cervical cancer, neuroblastoma, Rosai–Dorfman disease, plexiform neurofibroma

## Abstract

Due to the complex anatomy of the pelvis, various tumors may arise in this region. Some of these tumors are well known and have distinctive features that allow them to be identified by magnetic resonance imaging (MRI). These include sacrococcygeal teratoma (SCT), the most prevalent congenital tumor in children, often diagnosed prenatally and most frequently occurring in this anatomical location, and ovarian teratoma, which in its mature form is the most common ovarian neoplasm in children and adolescents. Additionally, rhabdomyosarcoma (RMS), commonly found in the bladder in both genders and in the prostate in males, and Ewing sarcoma (ES), affecting the flat bones of the pelvis, are relatively common tumors. In this study, selected atypical pelvic tumors in children are presented. Most of them are tumors of the reproductive system, such as cervical cancer, small cell neuroendocrine carcinoma of the ovary, ES/primitive neuroectodermal tumor (PNET) of the ovary, diffuse large B-cell lymphoma (DLBCL) of the ovaries and ovarian Sertoli–Leydig cell tumor (SLCT) with RMS due to DICER1 syndrome. Additionally, tumors originating from the nervous system, including neuroblastoma (NBL) and plexiform neurofibroma (pNF), associated and not associated with neurofibromatosis type 1 (NF1), are discussed. Furthermore, Rosai–Dorfman disease involving the pelvic and inguinal lymph nodes is presented. By reviewing the literature and presenting our cases, we tried to find radiological features of individual tumors that would bring the radiologist closer to the correct diagnosis, ensuring the implementation of appropriate treatment. However, the MR images cannot be considered in isolation. Additional patient data, such as the clinical picture, comorbidities/syndromes, and laboratory test results, are necessary.

## Simple Summary

Apart from pelvic tumors with typical radiological appearance, such as sacrococcygeal and ovarian teratoma, and less typical, such as rhabdomyosarcoma and Ewing sarcoma, the unusual masses may arise in the pelvis in the pediatric age group and these include cervical cancer, ovarian small cell neuroendocrine carcinoma, Ewing sarcoma/primitive neuroectodermal tumor of the ovary, ovarian diffuse large B-cell lymphoma (DLBCL), ovarian Sertoli–Leydig cell tumor with rhabdomyosarcoma, neuroblastoma, plexiform neurofibroma, and Rosai–Dorfman disease. After ultrasound which is the first-line imaging modality, magnetic resonance imaging is usually used for further characterization and diagnosis. Description of these entities and of the radiological features of these tumors is meant to bring the radiologist closer to the correct diagnosis, ensuring the implementation of appropriate treatment.

## 1. Introduction

The complex anatomy of the pelvis allows for the development of various cancers in this area. Lesions may occur in any pelvic organ and system, e.g., in the urogenital tract, gonads, soft tissues, or bones [1]. Therefore, there is enormous histological diversity in these lesions.

Pelvic tumors in children do not pose a significant diagnostic problem due to their relatively typical radiological appearance, especially on MRI, and the frequency of their occurrence, including sacrococcygeal teratoma (SCT)—the most prevalent congenital tumor in children, often diagnosed prenatally and most frequently occurring in this anatomical location—and ovarian teratoma, which in its mature form is the most common ovarian neoplasm in children and adolescents. Additionally, rhabdomyosarcoma (RMS), in the bladder in both genders and in the prostate in males, and Ewing sarcoma (ES), affecting the flat bones of the pelvis, are relatively commonly found.

A particular diagnostic problem is posed by those tumors for which the location in the pelvis is not typical, as well as those that are extremely rare in the pediatric population and mainly occur in adults. Additionally, these tumors usually have non-specific clinical symptoms.

In this study, we will focus on the MRI findings of selected atypical pediatric pelvic tumors that we have encountered in our practice so far; we will look for features of these masses that can help radiologists make the correct diagnosis and, consequently, help physicians choose and promptly implement the appropriate treatment.

## 2. More Common Pelvic Tumors in Children

SCT is recognized as the most common congenital tumor, with the sacrococcygeal region being its most prevalent location (extracranial teratomas accounting for 23–29% of congenital tumors, with SCT accounting for 45% of extracranial teratomas [2]). These tumors are diagnosed prenatally on ultrasound (US) followed by MRI, facilitating a more precise characterization of the lesion and determining its origin point. The type of SCT can also be accurately determined by MRI, according to the location-based classification system of the American Academy of Pediatric Surgery Section. Solid, solid-cystic, or exclusively cystic presentations are observed in these tumors, which may also feature calcifications/hemosiderin deposits and fatty tissue (Figure 1). Diffusion restriction is typically observed in solid components [2].

Ovarian tumors are generally uncommon in children; their estimated annual incidence is 2.6 per 100,000 girls and the tumors are typically benign [3]. However, ovarian teratoma is the most common ovarian tumor in children and adolescents—it accounts for over 50% of ovarian masses in women below 20 years of age [4]. It most often occurs in the mature form. The radiological appearance, both on US and MRI, is quite characteristic. This tumor usually presents as a cystic lesion with other features that are observed with varying frequency. The most common ones include the following: (1) the Rokitansky nodule—a mural solid nodule that protrudes into the cyst, with variable contrast enhancement; (2) fat in the tumor with hyperintensity on T1-weighted images (T1WI), signal drop on fat-saturated T1WI, and chemical shift artifact; and (3) keratinoid material that shows a low signal on T1WI, a high signal on T2-weighted images (T2WI), and marked diffusion restriction [5] (Figure 2).

RMS is the most common sarcoma arising from soft tissues in the pediatric population [6] and accounts for approximately 3% of new cancer cases in children aged 0–14 years per year [7]. Approximately 15–20% of these tumors are located in the urogenital system, where the urinary bladder and prostate are the most common origin point, followed by the vagina and uterus [8]. Sarcomas (mainly RMS) are the most common malignant vaginal, cervical, and uterine tumor type in girls [9]. Unfortunately, these locations are one of the unfavorable sites for RMS, which is associated with a worse prognosis. These tumors are usually large at diagnosis. Like RMS in other locations, they show intermediate signal intensity on T1WI and T2WI, diffusion restriction, and enhancement after contrast administration (Figure 3).

Bone tumors (OS and ES) constitute 4.7% of all cancers in children aged 0–14 years and 7·8% in those aged 15–19 years [7,10]. ES is the second most common malignant bone tumor in children after osteosarcoma (OS) and the most common pelvic bone tumor below 20 years of age [11,12]. A disproportionately large soft tissue component in relation to the bone infiltration itself is a characteristic feature of this tumor, which occurs in more than half of cases [12] (Figure 4). Histologically, the tumor is a small round blue cell tumor, translating into vivid diffusion restriction and prominent contrast enhancement on MRI.

## 3. Atypical Pelvic Tumors in Children

The probability of diagnosis depends—among others—on the age of patients. There are different typical diagnoses for neonates, children, and adolescents. Nevertheless, even with a final diagnosis of a typical tumor, we may be surprised by the location of a tumor in an unusual place. We may also be surprised by a histopathological diagnosis atypical for the age group. This section is devoted to such situations.

### 3.1. Small Cell Carcinoma of the Ovary, Hypercalcemic Type

Small cell carcinoma of the ovary, hypercalcemic type (SCCOHT), is a rare and aggressive cancer, constituting less than 0.01% of all ovarian cancers. It is often associated with paraneoplastic hypercalcemia, and the prognosis is extremely poor, regardless of the disease stage [13,14,15]. SCCOHT is classified as a miscellaneous tumor according to the 2014 World Health Organization (WHO) Classification of Cancers of the Female Reproductive Organs [16].

#### Case Report

*A 10-year-old patient complained of decreased appetite, polydipsia, vomiting, constipation, weight loss of 4–5 kg, and lower abdominal pain for about a month. Enlarged abdominal circumference with palpable resistance in the abdomen was found on physical examination and an increased total calcium level of 3.08 mmol/L was revealed (normal < 2.7).*

*US showed a large solid tumor with a maximal diameter of 150 mm, volume 906 mL, with areas of necrosis in the mid abdomen. Free fluid in the peritoneal cavity and peritoneal thickening were also found. The ovaries could not be visualized.*

*MRI confirmed an extensive mass with central necrosis, marked diffusion restriction, and contrast enhancement of the solid part (Figure 5). Numerous peritoneal and omental implants were found as well as large amount of fluid in the peritoneal and pleural cavities. The right ovary was not visible, suggesting that it was the source of the pathological mass.*

*The patient underwent surgery. The tumor, along with the right ovary and fallopian tube, were resected, and the thickened peritoneum and greater omentum were biopsied. Histopathological examination revealed disseminated ovarian small cell carcinoma of the hypercalcemic type.*

*Due to the presence of metastases, salvage chemotherapy was implemented and resulted in only transient clinical improvement. Five months after diagnosis, the patient died due to progression of the disease.*

SCCOHT mainly occurs in adolescents and young women, with an average age at diagnosis of 23.9 years [13,14]. Its occurrence in children is very rare, with only a few cases reported so far [17] and the youngest reported case at 12 months of age [13].

The pathogenesis mainly involves sporadic or germline mutations in SMARCA4/BRG1 and/or SMARCA2/BRM [16]. In our patient, as in almost all cases of SCCOHT, there was a complete loss of SMARCA4 expression [13].

The clinical presentation is non-specific. The most common symptoms include abdominal pain and enlargement [17]. Approximately two-thirds of patients develop hypercalcemia, which is related to the secretion of PTHrP by the tumor [14]. Symptoms of hypercalcemia are rare and occur in approximately 5% of patients. Our patient experienced symptoms related to elevated calcium levels, such as polydipsia and constipation. Other symptoms associated with hypercalcemia include polyuria, bone pain, and muscle weakness [3,17]. Interestingly, calcium levels decrease after tumor removal, so it is considered a valuable indicator for monitoring the course of the disease, especially for the detection of recurrence [17].

SCCOTH occurs unilaterally in most cases, more often in the right ovary, as in our patient [14,16]. The tumor is typically sizable, with its largest dimension ranging from 6 to 25 cm [13]. Similarly to the clinical symptoms, the radiological picture is not specific and may resemble other ovarian cancers [17]. The patients described in the literature have undergone US, computed tomography (CT), or MRI. The majority of these studies demonstrated an extensive, heterogeneous pelvic mass with or without central necrosis or calcifications, accompanied by fluid in the peritoneal cavity and/or pleural cavity [13,14,17,18,19,20,21,22]. Among these reports we identified two cases in which the MRI appearance of the tumor, characterized by extensive central necrosis, was comparable to the imaging findings in our patient [14,19].

Omental and peritoneal involvement were already present in our patient at diagnosis. Similar pediatric cases have been described in the literature [20,21,22]. Additionally, there have been reports of girls with metastases in the lymph nodes (para-aortic, external iliac) [20,22]. Other sites of SCCOHT metastasis included the lung, liver, pancreas, bladder, mediastinum, bone, spleen, cerebellum, vagina, and breast [16].

The FIGO classification, similar to that used for ovarian cancer, is applied to SCCOHT to determine prognosis. Tumors at the FIGO IA stage have the best prognosis, with a five-year overall survival rate of only 33%. Factors indicating a poor prognosis include age younger than 30, elevated calcium levels, tumors larger than 10 cm, and the presence of large cells [16]. Unfortunately, our patient exhibited three out of these four unfavorable factors—age 10, largest tumor size >17 cm, and elevated total calcium level. Additionally, she had peritoneal and omental metastases at diagnosis, indicating FIGO stage IIIc. This group’s overall five-year survival is estimated at 18% [13].

Therapeutic approaches to SCCOHT vary. The most frequently chosen treatment method is surgery, specifically fertility-conserving therapy such as unilateral salpingo-oophorectomy, which might be preferable for young patients. This is typically followed by adjuvant chemotherapy, which was used in our patient. Chemotherapy initially also resulted in a reduction in omental and peritoneal infiltrates, which gave the patient a chance for another surgical procedure to remove these changes, but further progression of the disease with a marked increase in infiltrates made this impossible.

### 3.2. Ewing Sarcoma/Primitive Neuroectodermal Tumor of the Ovary

The same histology, composed of small round blue cells, characterizes the Ewing sarcoma (ES) family of tumors. Additionally, a common chromosomal rearrangement, t(11;22)(q24;q12), is shared by them. This family encompasses ES of bone, extraskeletal ES, Askin tumors (ES of the thorax), and primitive neuroectodermal tumors (PNETs) [23]. Most cases of tumors from this family located in the gynecological system are PNETs, which are extremely rare lesions, with the ovary being the most common location, followed by the uterine corpus [24]. In the literature, we found only one case of extraskeletal Ewing’s sarcoma located primarily in the ovary in an 18-year-old woman. Typically, this type of tumor is located in the chest wall, paravertebral region, or lower limb [25].

#### Case Report

*A 10-year-old patient, 22 months after completing oncological treatment for a left ovarian tumor, was admitted to our center for further diagnosis and treatment of a sclerotic lesion in the right ilium, which was ultimately considered a bone island. She had a history of left ovary and fallopian tube resection due to PNET/extraskeletal ES. She underwent chemotherapy, teleradiotherapy (RTX) to the pelvic area, and a boost to the tumor area. She also underwent bone marrow stem cell separation. Unfortunately, there is no available history of clinical symptoms before the diagnosis.*

*A baseline pelvic MRI performed at the patient’s treatment center was provided for further comparison and showed a large pelvic and abdominal mass with a greatest diameter of 160 mm, accompanied by free fluid. The tumor had a solid-cystic structure with thick septations in the cystic part. The solid part showed substantial diffusion restriction and marked enhancement after intravenous gadolinium administration (Figure 6). The lesion was compressing adjacent organs but without any apparent signs of infiltration. The ovaries were not visible separately.*

Based on cases found in the literature, the age of the patients with PNET of the ovary ranges from 13 to even 79 years, but the majority are girls and women of premenopausal age [26].

As in our patient, the tumor usually affects only one ovary, although synchronous involvement of both ovaries may occur [27]. Despite the large size of the tumor at diagnosis (most cases were >10 cm in the largest size, as in our patient), the clinical symptoms are highly non-specific. These include abdominal/pelvic pain, mass in the abdomen/pelvis, weight loss, bloating, irregular periods, abnormal vaginal bleeding, and back and lower limb pain [24,26,27,28,29].

The radiological appearance of these tumors is non-specific as well. Therefore, they may be incorrectly interpreted as other, more common ovarian tumors, such as teratoma, ovarian carcinoma, or Krukenberg tumor [27,28,30]. In the cases described so far, three MRI patterns can be distinguished: (1) a large, solid, heterogeneous tumor with restriction diffusion and varying degrees of enhancement after gadolinium administration; (2) a mass consisting of thick-walled cystic lesions with numerous septa; (3) a combination of both patterns mentioned above [24,26,27,29]. The MRI of our patient’s tumor reflected the third pattern, with the simultaneous presence of solid and cystic parts. A common accompanying feature is ascites or hemoperitoneum and infiltration of adjacent organs, such as the omentum and intestines (small and large) [24,27,28]. At the time of diagnosis or during treatment, metastases are also frequently encountered, which significantly worsens the prognosis. They are commonly found in the lungs, bones, and bone marrow [28]. Fortunately, our patient did not have any invasion of adjacent structures or distant metastases.

There is no standard treatment for primary ovarian PNET. Surgical treatment remains the method of choice [26]. Fertility-sparing surgery (unilateral salpingo-oophorectomy) followed by adjuvant chemotherapy should be considered in young women [28]. Our patient additionally underwent radiotherapy.

### 3.3. Diffuse Large B-Cell Lymphoma of the Ovaries

Lymphomas affect 5.3% of children aged 0–4 years, and 22.5% of those aged 15–19 years. In children aged 0–14 years, lymphomas are the third leading cancer in the world, after leukemia and central nervous system tumors, and are the most common cancers in adolescents aged 15–19 years, [10]. Nevertheless, lymphomas in the genital tract are rare, with the ovary being the most common location. They may occur as a primary form in the ovary or secondarily involve the ovary as part of nodal disease [31]. Primary ovarian non-Hodgkin lymphomas (NHLs) denote an extranodal lymphoma in one or both ovaries without evidence of involvement of any other lymph node region or extra-lymphatic organ [32]. They account for 0.5% of all NHLs and 1.5% of all ovarian tumors [33]. The most common histological type of NHL in the ovary is diffuse large B-cell lymphoma (DLBCL), followed by follicular lymphoma; Burkitt lymphoma (BL)—most common in the pediatric population; and mucosal-associated lymphoid tissue lymphoma (MALToma) [31].

#### Case Report

*A 17-year-old patient was admitted due to enlarged abdominal circumference, abdominal pain, and constipation persisting for three months. Peripheral lymph nodes were not enlarged. Laboratory tests revealed increased concentrations of CA-125, 860 U/mL, and lactate dehydrogenase (LDH), 3096 U/L (normal ranges up to 35 U/mL and 670 U/L, respectively), along with a significantly reduced level of iron in the serum. US and CT revealed solid nodular masses of various sizes, filling in all the recesses of the peritoneal cavity. Diagnostic laparoscopy confirmed extensive infiltrates in the greater omentum. Histopathological examination revealed a DLBCL Burkitt-like.*

*Due to atypical spread and elevated CA-125 levels, pelvic MRI was performed and showed two large ovarian masses in contact with each other, well-circumscribed, heterogeneous, and with more hyperintense central parts on T2WI. Each mass had small, spherical fluid spaces, corresponding to ovarian follicles on the periphery, and a hypointense rim constituting its capsule. At the border of the field of view, extensive omental infiltrates were visible, which—similarly to ovarian masses—were heterogeneously enhanced after contrast medium administration (Figure 7).*

*Chemotherapy resulted in the regression of the peritoneal implants, with the ovaries remaining slightly enlarged post-treatment but regaining their typical follicular structure. The patient remains in remission of the disease.*

An important but challenging task is to differentiate between primary and secondary involvement of the ovary by lymphoma. Both processes differ in prognosis; primary ovarian lymphoma is associated with a better prognosis, and the 5-year overall survival rate is 70%, while for secondary involvement, it is 59.3% [31]. Fox et al. proposed criteria for primary ovarian lymphoma, according to which lymphoma is considered primary if, at the time of diagnosis, it is limited to the ovaries, no abnormal cells are found in the blood or bone marrow, and if dissemination occurs, a break of several months is required between the occurrence of changes in ovaries and dissemination [33]. In our patient’s case, due to the advanced disease at the time of diagnosis, it was not possible to determine whether the process in the ovaries was primary or secondary.

The histological type of the tumor is considered the most important prognostic factor; those derived from B cells are characterized by longer survival [34]. In addition, the level of the tumor marker CA-125 is also considered a predictive index. Approximately 45% of patients have elevated CA-125 levels, and this is usually associated with more advanced disease, poor response to treatment, and poor prognosis [31,32]. The same applies to elevated LDH levels, which are associated with a more aggressive course and worse survival outcomes [33]. This is confirmed in the case of our patient, where, at the time of diagnosis, the disease was already advanced with peritoneal infiltration, and CA-125 and LDH levels were significantly elevated.

The available literature shows that most cases of primary ovarian DLBCL occur between 20 and 40 years of age, with a median of 45 years [31]. To our knowledge, the case we present is one of the youngest cases of ovarian DLCBL described in the literature. Ovarian BL is more typical for the pediatric population and is usually diagnosed between 10 and 18 years of age. However, a 4-year-old girl with primary BL of the ovary and calcaneus has also been described [35].

Clinical symptoms of ovarian NHL are non-specific and most often include pain or discomfort in the abdominal cavity, enlarged abdominal circumference, and irregular bleeding [31,32]. B symptoms (fever, night sweats, or weight loss) may also occur (in 10–33% of cases) [31]. Our patient presented similar symptoms; no B symptoms occurred. Additionally, ovarian torsion and related symptoms may occur [36].

Ovarian lymphomas are often bilateral—in 38–71% of cases [37]. On imaging, they usually present as homogeneous, solid masses and central necrosis may occur. They usually exceed 5 cm. Small cysts arranged linearly around the periphery, corresponding to ovarian follicles, have also been described. In addition, a very suggestive symptom that should lead to the diagnosis of lymphoma is the marked enlargement of both ovaries that touch each other—“touching” large ovaries. Ascites usually do not accompany the masses. One study suggests that the absence of ascites in the presence of a bilateral, homogeneous ovarian mass is highly suggestive of lymphoma [38].

In the case of primary ovarian BL, the absence of other organs’ involvement is rare. The associated ascites, hepatosplenomegaly, dilatation of the bile ducts, omental infiltration, and thickening of the retroperitoneal space have been described [39,40,41]. Perhaps not without significance is the fact that our patient was diagnosed with a Burkitt-like lymphoma, and this may be why the peritoneum was also involved.

Multiagent chemotherapy is the treatment of choice, and not surgery, especially in the pediatric population [31]. The standard regimen is R-CHOP (rituximab, cyclophosphamide, doxorubicin, vincristine, prednisone).

### 3.4. Ovarian Sertoli–Leydig Cell Tumor with Rhabdomyosarcoma Due to DICER1 Syndrome

Sertoli–Leydig cell tumor (SLCT) is classified within the group of sex cord-stromal tumors, constituting less than 0.5–2% of all ovarian tumors [42,43]. Occurrence in the pediatric population is rare [43]. The classification is performed using degrees and characteristics of differentiation; according to the 2014 World Health Organization (WHO) guideline, they can be divided into well-, moderately, and poorly differentiated, heterogeneous, and reticular tumors [42]. Approximately 20% of moderately and poorly differentiated tumors, as well as those with retiform patterns, contain heterologous, mesenchymal, and/or epithelial elements [44,45]. The most common heterologous element is the mucinous epithelium of enteric type; five percent contain mesenchymal elements [45]. To date, approximately 20 cases of SLCT with elements of rhabdomyosarcoma (RMS) in the ovary have been described, some of which were associated with DICER-1 syndrome [43,45,46].

#### Case Report

*An 11-year-old patient, after treatment of pleuropulmonary blastoma completed seven years earlier, presented to the hospital with abdominal pain and a palpable mass. US revealed a large, solid pelvic tumor up to 165 mm in diameter, with cystic foci. This mass was pressing on the urinary bladder and the right ureter, causing dilatation of the pelvicalyceal system of the right kidney. Free fluid was visible in the abdominal cavity. The uterus and ovaries could not be visualized.*

**Figure 8:**
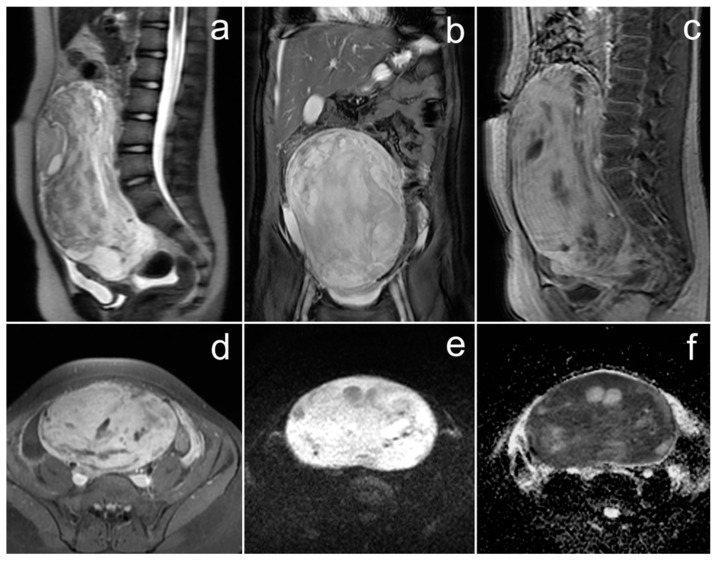
A Sertoli–Leydig cell tumor of the right ovary with rhabdomyosarcoma elements on MRI (**a**–**f**). (**a**)—FSE/T2; (**b**)—FIESTA; (**c**,**d**)—post-contrast LAVA/T1 WATER; (**e**)—DWI; (**f**)—ADC map.

**Figure 1:**
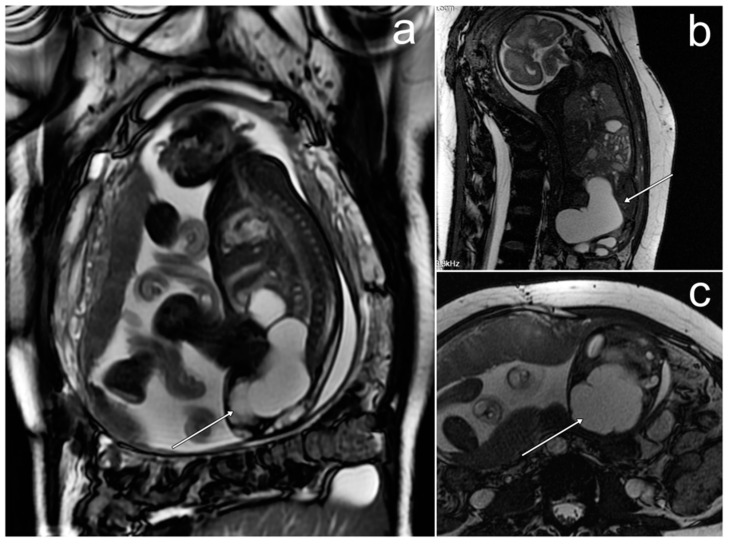
A fetal MRI. A singleton pregnancy, 29th gestational week (GW). A predominantly cystic sacrococcygeal teratoma, mainly located outside the body, a minor part of it in the pelvis, adjacent to the sacrum—type III, according to the classification of the Section of Pediatric Surgery of the American Academy (**a**–**c**, arrows). (**a**–**c**)—2D/FIESTA.

**Figure 2:**
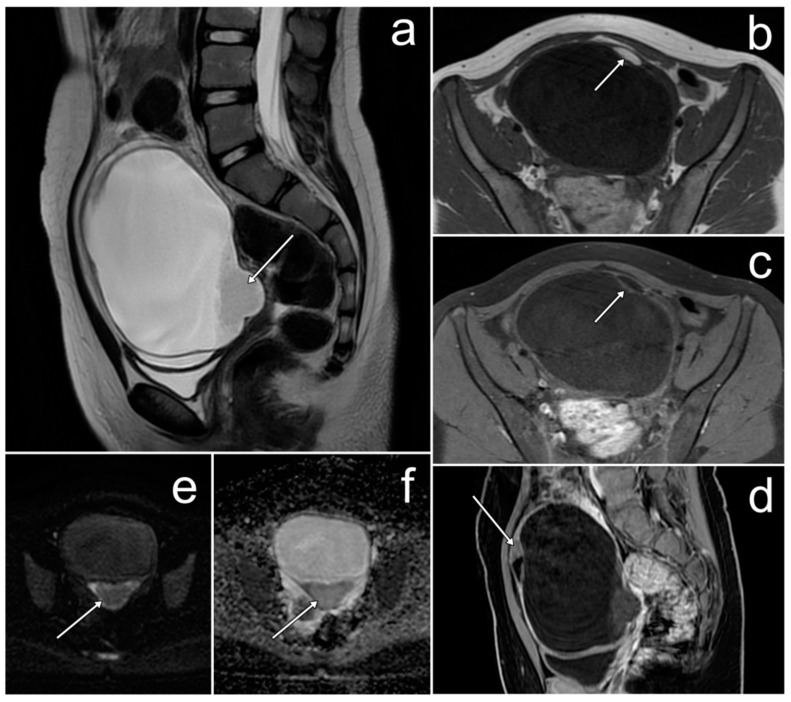
An 11-year-old girl with a giant cystic teratoma of the right ovary (**a**–**f**). Typical features of the lesion are present—a gravitationally arranged level of keratinoid material, which shows peripheral diffusion restriction (**a**,**e**,**f**, arrows), a Rokitansky nodule that poorly enhances after the administration of contrast agent (**d**, arrows), and a fatty element in the wall (**b**,**c**, arrows). (**a**)—FSE/T2; (**b**)—DIXON/T1 InPhase; (**c**)—DIXON/T1 WATER; (**d**)—post-contrast LAVA/T1 WATER; (**e**)—DWI; (**f**)—ADC map.

**Figure 3:**
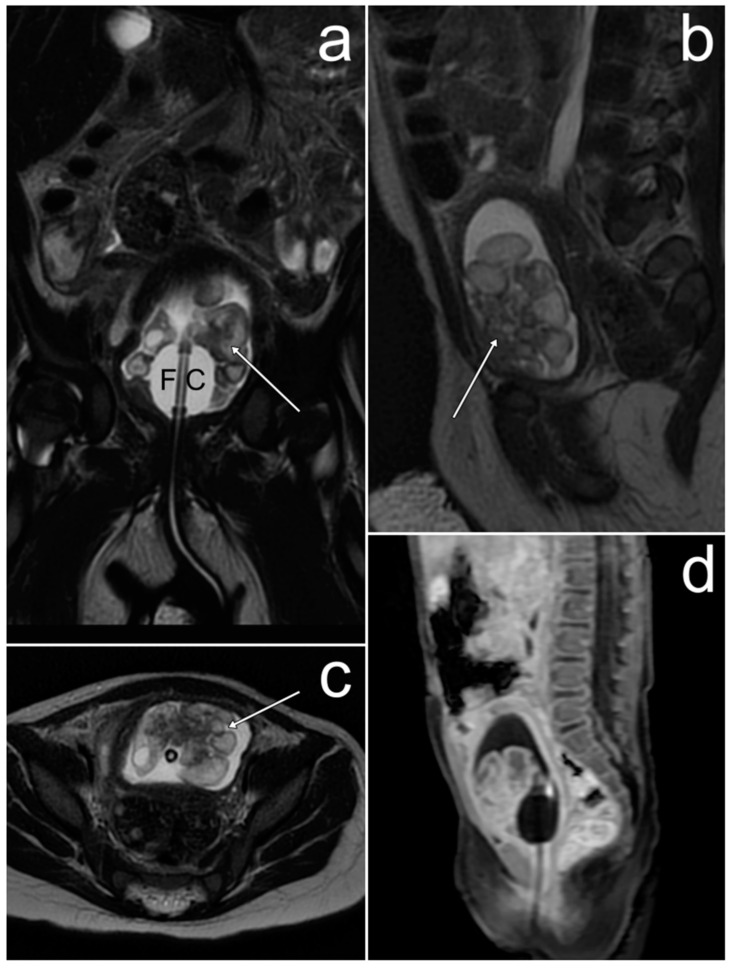
A 1.5-year-old girl with rhabdomyosarcoma of the bladder. Botryoidal masses in the lumen of the urinary bladder (**a**–**c**, arrows). FC—foley catheter. (**a**–**c**)—FSE/T2; (**d**)—post-contrast LAVA/T1 WATER.

**Figure 4:**
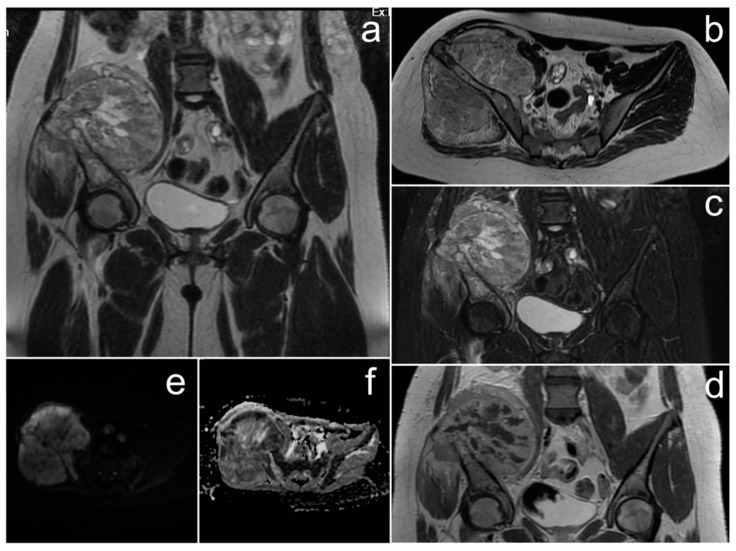
A 13-year-old girl with Ewing sarcoma of the right iliac wing with a giant soft tissue tumor on both sides of the bone (**a**–**f**). (**a**,**b**)—FSE/T2; (**c**)—STIR; (**d**)—post-contrast FSE/T1; (**e**)—DWI; (**f**)—ADC map.

**Figure 5:**
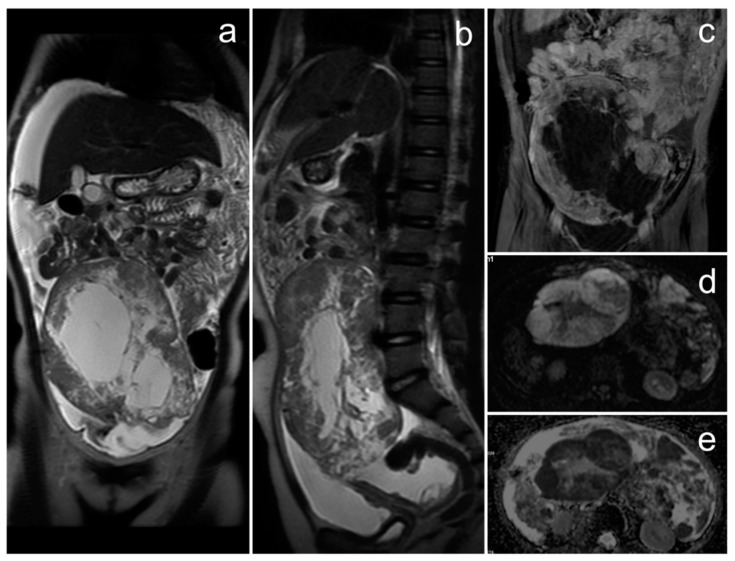
The MRI of a patient with small cell carcinoma of the ovary, hypercalcemic type (**a**–**e**). (**a**,**b**)—FSE/T2; (**c**)—post-contrast LAVA/T1 WATER; (**d**)—DWI; (**e**)—ADC map.

**Figure 6:**
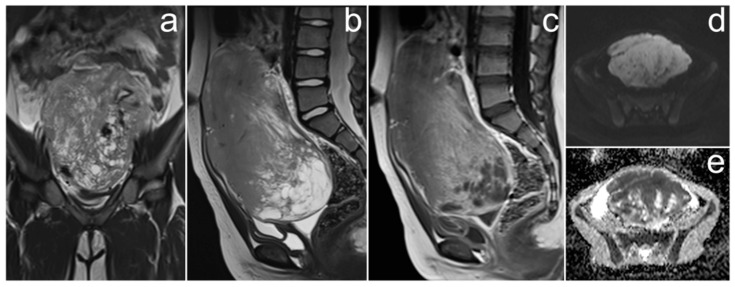
The MRI of primitive neuroectodermal tumor/extraskeletal Ewing sarcoma of the left ovary in a 10-year-old patient (**a**–**e**). (**a**,**b**)—FSE/T2; (**c**)—post-contrast FSE/T1; (**d**)—DWI; (**e**)—ADC map (images courtesy of Prof. K. Jonczyk-Potoczna, Poznan University of Medical Sciences).

**Figure 7:**
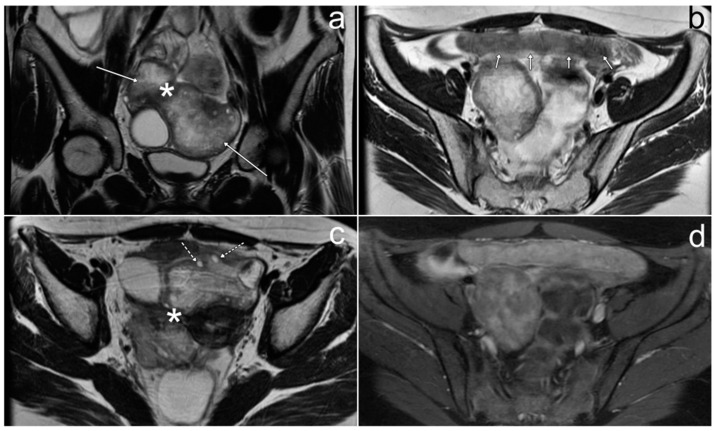
The MRI showing diffuse large B-cell lymphoma of both ovaries and peritoneal infiltrates (**a**–**d**). Enlarged ovaries touching each other ((**a**,**c**), long arrows, asterisks). Ovarian follicles at the periphery of masses ((**b**), dotted arrows). Peritoneal infiltration (**b**, short arrows). (**a**–**c**)—FSE/T2; (**d**)—post-contrast LAVA/T1 WATER.

*On MRI the maximum diameter of the well-circumscribed pelvic and abdominal tumor was 177 mm. The tumor was heterogeneously solid, with small fluid spaces and areas of hemorrhage. After the administration of gadolinium, it showed heterogeneous enhancement (Figure 8). The uterus, urinary bladder, and intestines were displaced and compressed, as well as the ureters, which resulted in bilateral dilatation of the pelvicalyceal systems. The ovaries were not visualized.*

*After biopsy, a mixed Sertoli–Leydig cell tumor with rhabdomyosarcoma elements was diagnosed. After chemotherapy, partial tumor regression was achieved. Then, surgery was performed to remove the tumor along with the right ovary and right fallopian tube, and RTX was administered to the tumor bed.*

*During sonography of the cervical and subclavian vessels before the implantation of the vascular port, attention was drawn to at least five nodules in the thyroid gland. After a fine-needle biopsy, multinodular goiter was confirmed, and the thyroid gland was resected.*

*Currently, the patient is under the care of an oncology clinic.*

The above compilation of lesions in our patient constitutes DICER1 syndrome. It is a rare, genetically determined syndrome that predisposes young patients to the development of various benign and malignant tumors. These lesions include pleuropulmonary blastoma (PPB), pulmonary cysts, ovarian tumors (sex cord-stromal tumors [most often SLCT], and sarcoma), embryonal RMS (ERMS) of the cervix, multilocular cystic nephroma, and thyroid gland neoplasia (adenomas, thyroid cancer, multinodular goiter [MNG]) [42,43,47,48].

Ovarian sex cord-stromal tumors in patients with DICER1 syndrome typically occur in individuals under 40 years of age and are most often moderately differentiated SLCT [43,48]. SLCT is associated with increased testosterone secretion, leading to hyperandrogenism symptoms in 35% of patients, including oligomenorrhea, amenorrhea, breast atrophy, acne, voice raucity, clitoromegaly, and hirsutism. In half of the cases, there are no hormonal manifestations, and symptoms are limited to those of the presence of a tumor in the abdomen/pelvis, such as mass effect, pain or discomfort, ascites, and tumor rupture [42,43]. Our patient’s testosterone level was normal, and no symptoms of virilization were present.

DICER-1 mutation is also associated with a more frequent occurrence of RMS, primarily of the embryonic type (ERMS), most often located in the cervix [43]. As in our patient, RMS elements may also coexist with SLCT in the ovary. The presence of sarcomatous elements is associated with a worse prognosis and a higher risk of recurrences after SCLT treatment [43,45,46,49].

The radiological appearance of SLCT may be variable [3]. It may occur as a solid, solid-cystic, or exclusively cystic tumor. The latter is rarely seen, but the solid-cystic form is identified in approximately 60% of cases. Depending on the presence of fibrous stroma, a heterogeneous signal is shown by solid parts on T2WI. A low signal on T1WI and a high signal on T2WI are shown by cystic parts, but bleeding may occur, which is manifested by an increased signal on T1WI. Solid elements usually show strong enhancement in the arterial phase due to hypervascularity. In subsequent phases of the contrast examination, the fibrous stroma is gradually enhanced [50,51]. Our case presents the most common solid-cystic form with areas of hemorrhage. These tumors have usually been described as large so far; in two studies, sizes ranging from 1.5 to 30 cm have been reported [49]. In our patient, the largest tumor size was over 17 cm.

To the best of our knowledge, no studies describing cases of the coexistence of SLCT and RMS in the ovary have focused on the features of this combination on imaging studies. We believe that the presence of RMS elements does not significantly affect the appearance of the tumor. Therefore, it should be based on the features of SLCT itself.

The basis of treatment is surgery with the removal of the ovary and fallopian tube. There is no standard chemotherapy regimen for advanced or recurrent SLCT with RMS [43]. Our patient received chemotherapy for RMS and achieved a good response to this treatment. The literature also mentions the effectiveness of such treatment [49].

### 3.5. Primary Ovarian Angiosarcoma with Peritoneal Metastasis

Angiosarcoma (AS) is a sporadic malignant tumor accounting for <1% of soft tissue sarcomas in children [52]. It can be located anywhere in the body and originate from different organs. Most often, however, it is located in the soft tissues of the head and neck (approx. 50% of cases), while involvement of the genital system is extremely rare. AS accounts for approximately 4% of uterine cancer and 1% of ovarian cancer [53]. In the ovary, it may occur as a pure sarcoma or in combination with other ovarian tumors such as teratoma, mucinous cystadenocarcinoma, and dermoid cyst. So far, 53 cases of this primary ovarian AS (OAS) have been described [54].

#### Case Report

**Figure 9:**
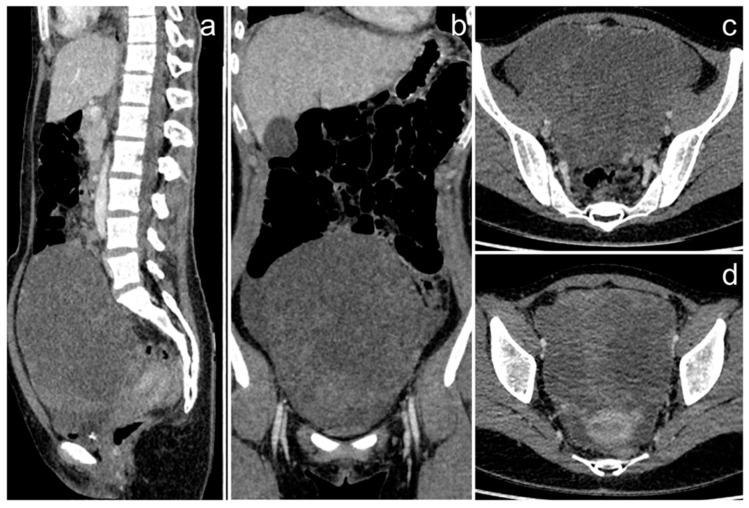
The CT exam of the primary ovarian angiosarcoma (**a**–**d**). Extensive, cystic-solid mass in the pelvis (images courtesy of Dr. M. I. Furmanek, Medical University of Warsaw).

*A 16-year-old patient was transferred from another center to continue treatment of a disseminated left ovarian tumor. Initial CT outside our center revealed a cystic-solid pelvic mass with thick, irregular septa, contrast enhancement and a network of pathological vessels (Figure 9), and free fluid. The largest dimension of the tumor was 166 mm.*

**Figure 10:**
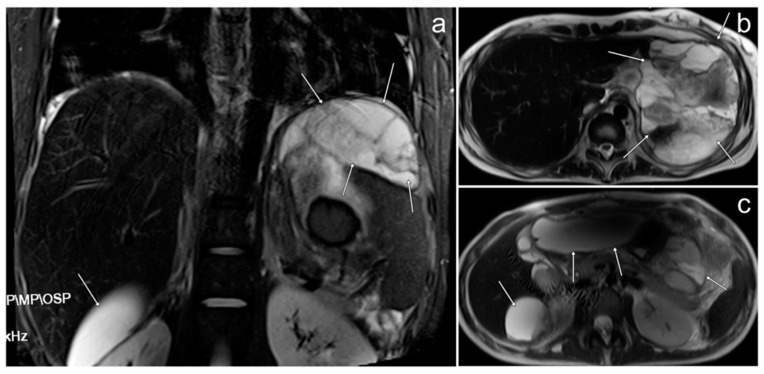
The MRI of the peritoneal metastases of ovarian angiosarcoma (**a**–**c**, arrows). (**a**)—STIR; (**b**,**c**)—FSE/T2 (images courtesy of Dr. M. I. Furmanek, Medical University of Warsaw).

*The tumor was resected along with the left adnexa, greater omentum, para-aortic lymph nodes, and appendix. Just before transfer to our center, approximately two months after diagnosis, whole-body MRI was performed, which revealed inhomogeneous cystic and solid lesions located mainly in the upper and mid abdomen: around the spleen, adjacent to the stomach, pancreas, and liver (Figure 10). Small enhancing nodules were also visible on the surface of the liver. There was a small amount of fluid in the left pleural cavity. All these changes resembled the primary tumor in their morphology and were consistent with metastases.*

*Pathological examination initially indicated an anaplastic variant of juvenile granulosa tumor with omental deposits, but the final diagnosis was angiosarcoma. Due to the advanced disease, the patient was qualified for systemic chemotherapy and targeted treatment with sorafenib, which initially resulted in partial regression of the changes and stabilization of the disease. After a year of treatment in our hospital, the patient’s parents changed the center, in which the abdominal lesions were resected. We have no data on further treatment. The patient died due to progression of the disease 19 months after diagnosis.*

OAS usually occurs in the premenopausal age; the average age at diagnosis is approximately 31–33 years, and the youngest described patients were 11 years old [52,53,55,56].

Clinical symptoms are non-specific. Initially, the tumor is asymptomatic, but if it reaches a significant size it has a mass effect with neurological symptoms as a result of nerve compression [53,55]. The most common symptoms are abdominal pain and distension [52,55]. Additionally, ascites may occur, which was also present in our patient, as well as hemoperitoneum [56].

There are no specific tumor markers that would help in the diagnosis. Pathological diagnosis is necessary and may be difficult due to the complex and diverse characteristics of the tumor [55]. OAS may histologically resemble other neoplasms, such as certain sex cord-stromal and germ cell tumors, malignant melanoma, poorly differentiated carcinoma, metastatic angiosarcoma, and benign hemangiomas [53,54]. This confirms the initial incorrect diagnosis in our patient, which suggested that the tumor was a lesion arising from the sex cord-stromal— juvenile granulosa cell tumor.

It is usually unilateral, with the predominant involvement of the right ovary. Bilateral ovarian involvement is rare [56]. The radiological appearance of our patient’s tumor corresponds with the images of previously described cases. According to one study, these tumors usually reach large sizes; the average size is 11.9 +/− 6.1 cm [51]. Our patient’s tumor size is within this average range. OAS presents mainly as lesions with cystic and solid elements in various proportions [52,53,55,56]. On MRI, it may show different signals; hyperintensity on T1WI is associated with hemorrhage, hyperintensity on T2WI with a cystic component, and hypointensity on T2WI with a fibrous component. After the administration of gadolinium, solid elements usually show intense enhancement, which is related to the dense vascularization of the tumor [57]. The MRI features mentioned above can be found in the metastatic lesions in our patient.

Metastases occur in >50% of cases [54,57]. They can be located in the lungs, lymph nodes, liver, bones, intestinal wall, and peritoneum [58,59,60,61,62]. Our patient developed multiple peritoneal metastases.

The main treatment options are surgical debulking, postoperative chemotherapy, and radiotherapy [56]. The prognosis is poor and the 5-year survival rate is 10–35% [52]. The most important factor influencing the prognosis is clinical staging [56].

### 3.6. Cervical Cancer

Cervical cancer is sporadic in the pediatric population [63]. It most often occurs in the group of young women; according to The Surveillance, Epidemiology, and End Results (SEER), it is most often diagnosed in women between 33 and 40 years of age; the average age at diagnosis is 50 years.

There are many histological types of this cancer. In the adult female population, it is most often squamous cell carcinoma, strongly associated with human papillomavirus (HPV) infection.

Adenocarcinoma is the most common histological type reported in the pediatric and adolescent population, usually associated with intrauterine diethylstilbestrol (DES) exposure [63]. The remaining cases described are single cases of squamous cell carcinomas or in patients with HIV [64,65,66].

**Figure 11:**
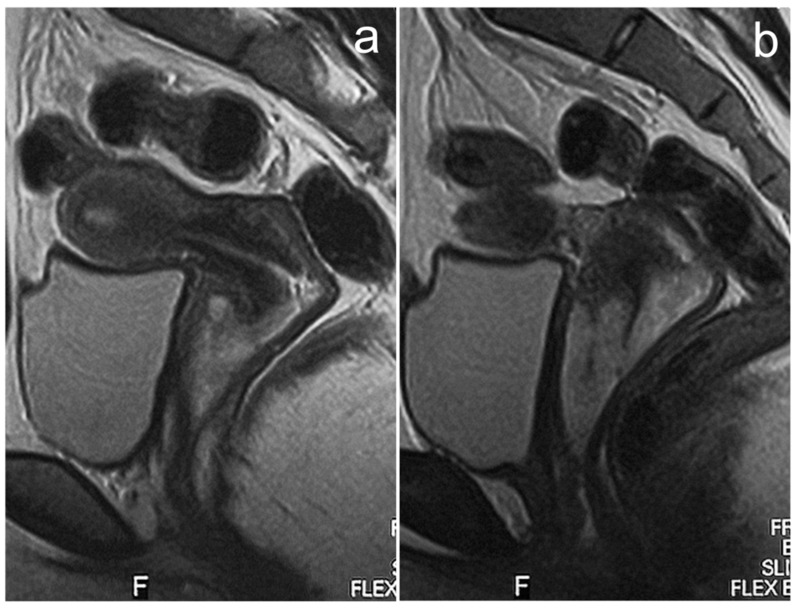
MRI of cervical cancer in 16-year-old girl (**a**,**b**). (**a**,**b**)—FSE/T2.

In our material there is one case of a 16-year-old patient with cervical cancer diagnosed in 2002. Only T2-weighted MR images are available, which show an exophytic pathological mass arising from the cervix, infiltrating the vaginal vaults, and occupying the upper two-thirds of the vagina (Figure 11). According to the FIGO classification, this corresponds to stage IIA. Unfortunately, we do not know the tumor histological type or the patient’s fate.

MRI is the examination of choice for staging and treatment planning, with the obligatory T2-weighted images and DWI sequence and optional contrast medium administration [67]. The radiological picture does not differ from that in adult women. The cervical tumor is best visible on T2-weighted images, showing higher signal intensity than the cervical stroma, and on DWI sequences. It may be an expansive or infiltrating lesion. According to the FIGO classification, the tumor size, parametrial invasion, vaginal involvement, bladder and rectal invasion, and the status of the lymph nodes are assessed [68].

### 3.7. Pelvic Neuroblastoma

Neuroblastoma (NBL) is a childhood neoplasm, the third most common after leukemia and brain tumors, and the most common extracranial solid malignant tumor [69,70]. It accounts for approximately 7% of new cancer cases in children aged 0–14 years per year [7]. It originates from the primitive cells of the sympathetic nervous system, most commonly in the adrenal glands. However, it can originate wherever sympathetic cells are present, from the neck to the pelvis. Nevertheless, pelvic location is uncommon; it is estimated that only 1–2.2% of neuroblastomas localize here, most often in the presacral area [70,71]. Some of these tumors may also originate from Zuckerkandl’s organ and, in this case, be located more anteriorly [70].

#### Case Report

*A 6-month-old girl presented to our center with a suspicion of a pelvic tumor. The clinical symptoms included urinary retention, gastrointestinal obstruction, perineal swelling, gaping anus, and paralysis of the lower limbs. US revealed a solid, heterogeneous tumor in the presacral area without detectable calcifications. The lesion was pressing against the rectum and uterus, but no enlarged lymph nodes were observed in the abdominal cavity or pelvis.*

**Figure 12:**
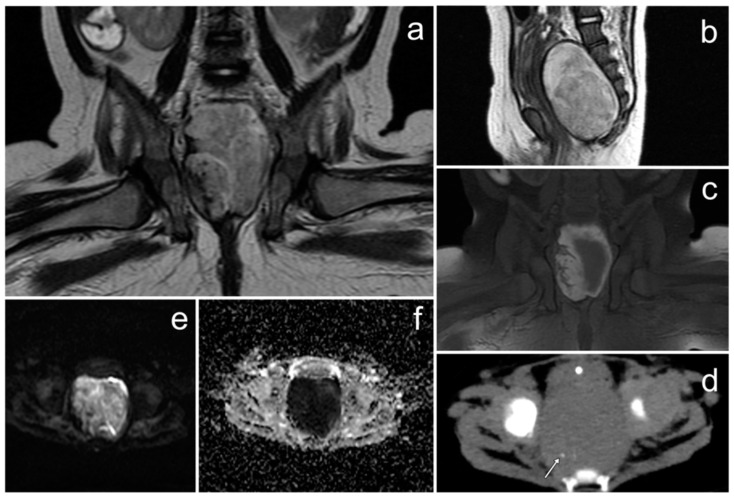
MRI of pelvic neuroblastoma (**a**–**c**,**e**,**f**). Small punctate calcification in tumor on CT scan (**d**, arrow). (**a**,**b**)—FSE/T2; (**c**)—post-contrast FSE/T1 FS; (**e**)—DWI; (**f**)—ADC map.

*Initially, the mass was suspected to be a sacral teratoma. Further diagnostic imaging with CT and MRI confirmed the presence of a pathological mass in the presacral area measuring up to 55 mm. The lesion contained small punctate and band-shaped calcifications. MRI showed a high T1 signal peripherally, while the central part was hypointense with no contrast enhancement (Figure 12). The mass was compressing adjacent organs and the S2–S4 nerve roots but did not penetrate into the spinal/sacral canal.*

*Several lymph nodes up to 6 mm in the short axis were visible near the large abdominal vessels.*

*The girl underwent surgery. Histopathological examination diagnosed the mass as an NBL. Chemotherapy was initiated due to the presence of a residual mass, which increased in size during subsequent follow-up examinations.*

*The patient is currently nine years after the completion of oncological treatment and comes to our center once a year for check-ups, including abdominal and pelvic US.*

The clinical symptoms of NBL depend mainly on its primary location, the impact the mass has on adjacent organs, and the presence of bone metastases or paraneoplastic syndrome [72]. In case of pelvic NBL, it is usually an asymptomatic palpable mass. Urinary retention, as seen in our patient, is one of the rarer symptoms of NBL in this location [71,73].

Imaging is initially based on US, but MRI, which does not use ionizing radiation, is a method that allows for accurate imaging of the tumor, its relationship to adjacent structures, and possible penetration into the spinal canal. Image-defined risk factors (IDRF) are used in preoperative staging; these are 20 risk factors assessed in imaging studies (CT/MRI) for multiple organ systems that help predict surgical treatment results and resection adequacy and provide initial risk stratification in combination with clinical data [74]. Concerning pelvic NBL, the relevant IRDFs are iliac vessel encasement and crossing of the sciatic notch by the tumor, which were not present in our patient [70]. In addition, whole-body MRI (WBMRI) is a valuable exam that probably increases diagnostic accuracy and allows for staging (e.g., determining localized disease or the presence of distant metastases) and monitoring treatment, together with metabolic studies such as metaiodobenzylguanidine (MIBG) scintigraphy and positron emission tomography (PET) [75].

Typically, these tumors show heterogeneous MRI signals due to calcification, hemorrhage, and necrosis. In the literature, variable signals on T1WI and T2WI are described [70]. In our patient, the lesion showed a peripherally high signal on T1WI, probably resulting from hemorrhagic changes, while the central part, which did not enhance after gadolinium administration, corresponded to necrotic changes. NBL is characterized by high cell density, which translates into strong diffusion restriction, visible as a high tumor signal on DWI sequences and low values of the apparent diffusion coefficient on ADC maps. Additionally, it is believed that ADC values allow the differentiation of NBL from two other tumors from the same family with a lower grade of malignancy—ganglioneuroma and ganglioneuroblastoma. According to one study, the average value of ADC in ganglioneuroma and ganglioneuroblastoma cases does not fall below 1.1 × 10^−3^ mm^2^/s, unlike in NBL [76]. In our patient’s case, the tumor’s average ADC value was approximately 0.4 × 10^−3^ mm^2^/s, which confirms these reports.

NBL in the pelvis has a much better prognosis than in other locations and is characterized by an excellent survival rate. However, it is associated with a higher risk of neurological complications related to tumor resection [73]. Our patient underwent surgery, which turned out to be non-radical, so additional chemotherapy was administered. She has completed treatment and currently does not report any disturbing symptoms.

### 3.8. Pelvic Plexiform Neurofibroma

Peripheral nerve sheath tumors (PNSTs) constitute a heterogeneous group with a broad spectrum of morphological features and biological potential. As the name suggests, they come from nerve sheaths outside the central nervous system. They vary from benign (schwannoma, neurofibroma [NF], perineurioma), through benign but potentially locally aggressive (plexiform NF [pNF]), to highly malignant (malignant peripheral nerve sheath tumors [MPNST]) [77]. The most common form in this group is NF, which occurs sporadically in approximately 90% of cases; in the remaining cases, especially when dealing with multiple lesions, it is associated with neurofibromatosis type 1 (NF-1). NF may occur in three forms: (1) localized, (2) diffuse, and (3) plexiform. The latter is pathognomonic for NF1 [78]. However, our material includes two NF-1 patients with pelvic (and abdominal) pNF, and, in the third one, pNF, interestingly, was not related to NF1.

#### 3.8.1. Case 1

*In a 17-year-old boy with NF-1, MRI of the lumbar–sacral spine revealed numerous oval or spherical-shaped lesions forming plexuses in the following areas: (1) the retroperitoneal space and pelvis, surrounding and modeling the rectum, urinary bladder, prostate, and seminal vesicles; (2) the intervertebral foramina and paraspinal regions along the spinal nerves; (3) the iliopsoas muscles, abdominal wall muscles, and dorsal and gluteal muscles; and (4) the subcutaneous tissue within the field of view.*

**Figure 13:**
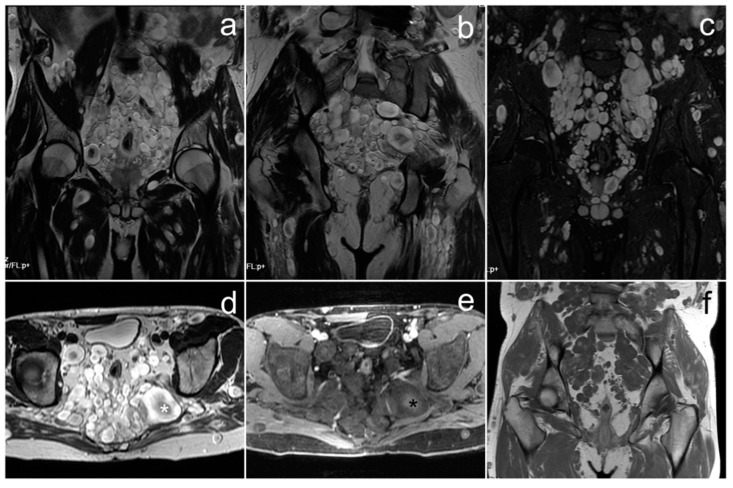
Pelvic MRI in Case 1 (**a**–**f**). In images (**d**,**e**), typical signs of neurofibroma are marked with asterisks: (**d**)—”target sign”; (**e**)—”reverse target sign”. (**a**,**b**,**d**)—FSE/T2; (**c**)—STIR; (**e**)—post-contrast FSE/T1 F; (**f**)—post-contrast FSE/T1 post-Gd.

*All the lesions had the same morphology. On T2-weighted images they appeared as targets with a hyperintense periphery and a hypointense center. Their diameters ranged from a few to 30 mm. After the administration of gadolinium, the central part of the lesions showed slight enhancement (Figure 13). None of the lesions demonstrated obvious diffusion restriction.*

#### 3.8.2. Case 2

*In a 17-year-old girl with NF-1, a follow-up US revealed heterogeneous pelvic masses, poorly demarcated from the surrounding normal organs, with no suspicious flow. The approximate dimensions of the area were 120 × 110 × 70 mm.*

**Figure 14:**
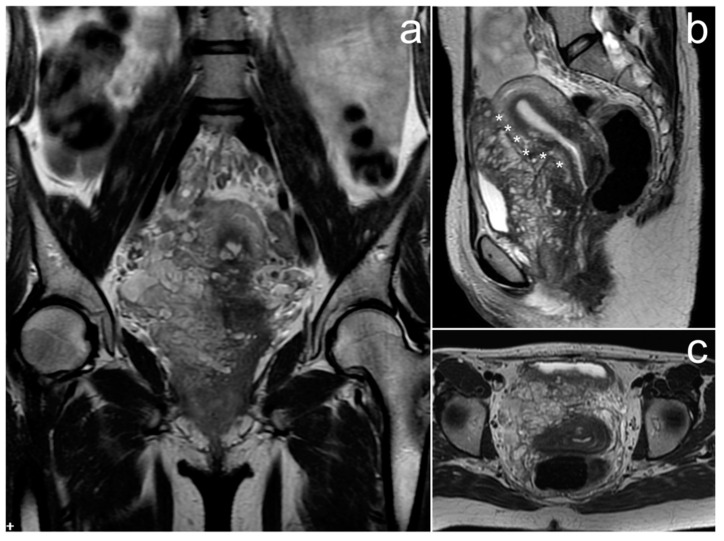
The pelvic MRI in Case 2 (**a**–**c**). The asterisks in (**b**) indicate infiltration of the anterior uterine wall and anterior cervical surface by plexiform neurofibroma. (**a**–**c**)—FSE/T2.

*Pelvic MRI was performed and showed an extensive abnormality surrounding the urinary bladder, uterus, ovaries, and rectum. The area consisted of small lesions, which on T2WI showed a low signal in the central part and a high signal in the periphery (fascicular sign). There was no boundary between the abnormality and the anterior wall of the uterus (body and cervix), as well as between the lesion and the posterior wall of the bladder, which was thickened and irregular (Figure 14), indicating infiltration.*

*The patient had neurofibromas in the intervertebral foramina in the lumbar and sacral spine on both sides, as well.*

*The patient remained under the care of the oncology clinic until early adulthood, without significant change on subsequent follow-up MRI examinations. The patient’s further clinical course is unknown.*

#### 3.8.3. Case 3

*An almost two-year-old patient diagnosed with cutaneous juvenile xanthogranuloma (JXG) presented to abdominal US without any clinical symptoms. The enlarged, heterogeneous prostate gland with a volume of approximately 24 mL was shown, as well as the irregularly thickened bladder wall, up to 9 mm, particularly on the right side.*

**Figure 15:**
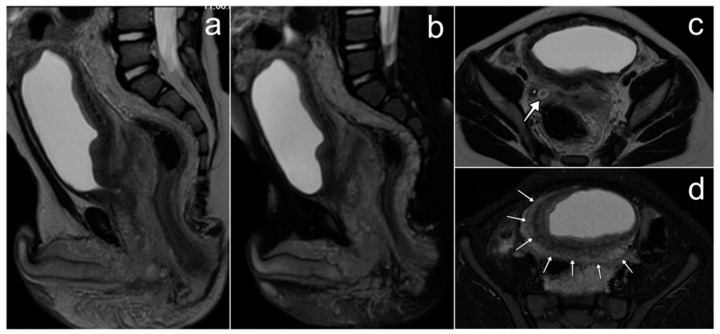
The pelvic MRI in Case 3 (**a**–**d**). A thick arrow in (**c**) marks the largest lesion forming plexiform neurofibroma with a typical “target sign”. Short arrows in (**d**) indicate the thickened, infiltrated urinary bladder wall. (**a**,**c**)—FSE/T2; (**b**,**d**)—STIR.

*The patient was referred to pelvic MRI, which confirmed the irregular thickening of the bladder wall (up to 17 mm) and revealed an extensive pathological area in the retrovesical and supravesical regions, extending perianally and to the penis and perineum. The mass was continuous with the outlines of the prostate gland (making the gland impossible to isolate) and the seminal vesicles. On T2WI, the entire pathological infiltrate consisted of tiny round lesions with fascicular sign (hyperintense on the periphery), giving the appearance of a “bag of worms” (Figure 15). It was biopsied, and histopathological examination revealed a small NF fragment. Based on histopathology and the MR exam, pNF was diagnosed.*

The patient remains under observation and the lesion is stable in follow-up exams.

NFs can occur at any age. The localized form most often affects adults aged 20 to 40, while the diffuse and plexiform forms are more characteristic of the pediatric population. Moreover, pNF is rare in children over five years of age, as confirmed by our Case 2 [78].

NFs most often occur in the skin and subcutaneous tissue; however, they can develop anywhere in the body [79]. Pelvic NFs are rare and mainly extraperitoneal [80]. They can occur either as isolated tumors or, in the case of NF1, where they are the most common type of neoplasm, in the gastrointestinal and urogenital systems [79,81].

Our third patient with JXG was not diagnosed with NF1. However, it should be noted that JXG occurs more frequently in NF1 than in the general population [82]. A high incidence of JXG is described in the literature in patients with NF1, especially in children under two years of age [83]. Additionally, involvement of the urinary bladder by pNF, as in the case of this patient, is pathognomonic for NF1 [84].

Due to distribution along the lumbosacral plexus, the most common locations are the paraspinal and presacral areas [84]. Of the pelvic organs, the urinary bladder is most commonly involved. In males, the prostate, seminal vesicles, and urethra, and in females, the uterus, vagina, or urethra may also be affected [81].

Localized NFs are usually asymptomatic. In case of pNF, clinical symptoms depend mainly on the location and size of the lesion. They extend along many nerve bundles, and those located deeper can cause pain, numbness, paresthesia, mass effect, and symptoms of spinal cord compression [78]. NFs affecting the pelvis may cause the obstruction and dysfunction of the gastrointestinal and urogenital tracts [80]. When the urogenital system is affected, it usually causes recurrent urinary tract infections and an urge to urinate [81].

On MRI, pNF is an extensive, infiltrative mass spreading along the nerve plexuses and showing a high signal on T2WI [84]. It is usually described as a lobular lesion, but in fact, it consists of numerous lesions affecting individual nerve bundles. These lesions show the “target sign” characteristic of neurofibroma on T2WI—with a hyperintense periphery, which reflects the mucosal component, and a hypointense center, which corresponds to the fibrous component. On T1WI, the lesion is usually hypointense in relation to the adjacent muscles. After gadolinium administration, a “reverse target sign” is visible, with enhancement of the center of individual lesions [85]. Similar images were found in our patients. In Case 1, the changes affecting individual nerve bundles were more significant and had a visible “target sign” and a “reverse target sign” after gadolinium administration. However, in Cases 2 and 3, the changes forming the infiltration were much more minor, and it was more difficult to notice these signs.

These changes usually do not show diffusion restriction; in our patients the average ADC value was >2.4 × 10^−3^ mm^2^/s.

From the oncological point of view, the possible malignant transformation of pNF is crucial; a malignant form, MPNST, causes death in up to 55% of patients with NF-1. This transformation occurs in approximately 8–13% of NF-1 patients [86]. Fortunately, certain MRI features make differentiating benign pNF from MPNST easier. The disturbing symptoms include an increase in lesion size; lesions >4.2 cm are considered highly suspicious [84]. Irregular shape, poor lesion delineation, intratumoral lobulation, heterogeneous signal intensity on T1WI, loss of the characteristic “target sign” on T2WI, cystic areas within the mass, and edema around the lesion should raise concern [86]. After gadolinium administration, enhancement is visible on the lesion’s periphery—the loss of the “reverse target sign“ [84]. Moreover, the presence of diffusion restriction with ADC < 1.0 × 10^−3^ mm^2^/s is a valuable feature indicating the malignancy of the lesion [87].

When we have a preliminary suspicion that the lesion may have transformed into a malignant form, fluorodeoxyglucose positron emission tomography (FDG-PET) should be performed, with increased tracer uptake confirming the suspicion [87].

Treatment involves the surgical removal of the lesions. When complete removal is not possible, even partial resection is preferred. In case of unresectable and progressive tumors, treatment with interferon was attempted [85]. Our patients were or remain under observation, and control tests are being performed. None of them had surgical treatment.

### 3.9. Rosai–Dorfman Disease Involving the Pelvic and Inguinal Lymph Nodes

Rosai–Dorfman disease (RDD)**,** also known as sinus histiocytosis with massive lymphadenopathy, is a rare non–Langerhans cell histiocytosis caused by the proliferation of histiocytes in the lymph nodes. Its etiology is unknown [88]. According to the Cleveland Clinic, its incidence is 1:200,000. The disease most frequently affects the cervical nodes, followed by the mediastinal, axillary, and inguinal nodes [89]. In approximately 43% of cases, the extranodal form occurs and most often affects the head, neck, skin, subcutaneous tissue, and bones. Both forms can occur independently, but enlarged lymph nodes accompany the extranodal form in 80% of cases [90].

#### Case Report

*An 8-year-old girl presented with a soft tissue tumor in the right groin, persisting for two months, without any other symptoms. A vascular lesion was initially suspected clinically, but US outside our center revealed package of lymph nodes.*

**Figure 16:**
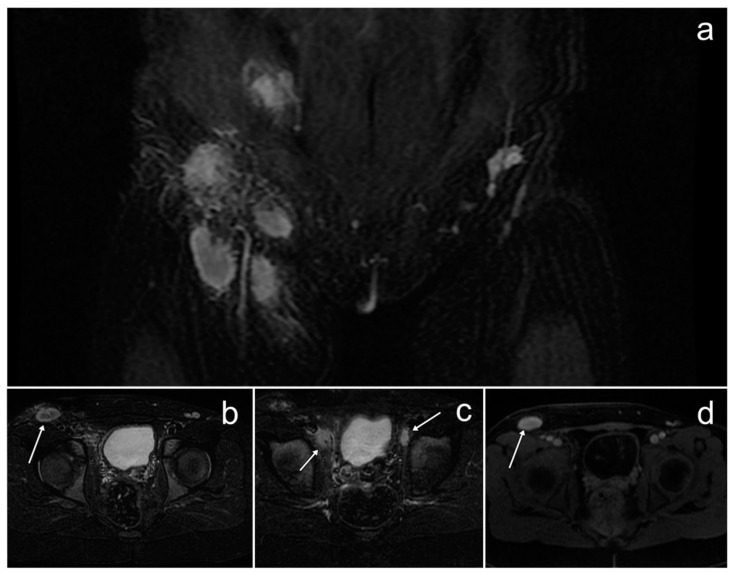
The inguinal and pelvic lymph nodes in Rosai–Dorfman disease on MRI (**b**–**d**, arrows). (**a**–**c**)—STIR; (**d**)—post-contrast LAVA/T1 WATER.

*On pelvic MRI, enlarged right inguinal lymph nodes up to 23 mm were revealed, as well as pelvic lymph nodes along the external iliac vessels on both sides up to 18 mm. These nodes had blurry boundaries but did not form packets. The surrounding adipose tissue showed a heterogeneous signal intensity, indicating reactive changes. After gadolinium administration, the nodes showed strong contrast enhancement (Figure 16).*

*Additionally, the examination revealed venous system anomalies, including the absence of the common iliac veins and the inferior vena cava below the confluence of the renal veins. The external and internal iliac veins were connected and drained into the paravertebral veins. Furthermore, dilated venous plexuses of the left parametrium, the left ovarian vein, and the left internal iliac vein were visible.*

*Pathological examination of one of the enlarged right inguinal lymph nodes revealed sinus histiocytosis. The patient remained under the care of the oncology clinic and underwent follow-up US and MRI scans, which showed that the enlarged lymph nodes were stable. We have no information about the girl’s treatment and current condition.*

The disease primarily affects children and young adults, with a mean age of 20 years. The clinical presentation is non-specific and diverse. General symptoms may include fever, weight loss, night sweats, a palpable mass in the subcutaneous tissue, bone pain, cough, or shortness of breath. The clinical course depends on the form of the disease. In isolated nodal disease, remission occurs in 20–40% of cases, sometimes spontaneously. In extranodal disease, the course is chronic with recurrences. Mortality in the extranodal form reaches up to 40%, particularly when the lower respiratory tract, liver, or kidneys are affected [90].

The presence of this disease in the abdominal cavity and pelvis is extremely rare and mainly occurs in the nodal form, with inguinal lymphadenopathy in approximately 44% of cases and pelvic lymphadenopathy. The extranodal form in the pelvis is much rarer and may present as a presacral mass, which is a potential distinguishing feature. It may also involve pelvic organs such as the uterus, ovaries, scrotum, vessels, and ureters (with secondary hydronephrosis) [90]. In our case, it was a purely nodal form.

Imaging diagnostics initially rely on chest X-ray and abdominal US, while MRI plays the most crucial role in identifying, staging, and monitoring the disease. MRI typically reveals significantly enlarged lymph nodes in the nodal form and abnormal single or multifocal masses in the extranodal form [89,90]. These changes may displace or compress adjacent organs. The masses usually exhibit an isointense signal on T1WI and a hypointense signal on T2WI [89]. They often show diffusion restriction, which can disappear in response to treatment [90]. After gadolinium administration, the masses display homogeneous enhancement [89].

The nodal form may resemble other diseases involving enlarged lymph nodes, primarily neoplastic diseases such as lymphoma, Kaposi sarcoma, metastases, and malignant histiocytosis, as well as inflammatory diseases and both infectious (tuberculosis) and non-infectious granulomatous diseases (Castleman’s disease, Wegener’s granulomatosis, sarcoidosis) [90].

We found no association between sinus histiocytosis and venous system anomalies in the available literature.

Pediatric patients with RDD generally have a good prognosis, with treatments mainly focusing on controlling symptoms. Most RDD cases can be managed with regular observation. For symptomatic cases, oral steroids are effective in reducing the size of masses and are considered a first-line treatment. If steroids are ineffective, other options, such as immunotherapy, chemotherapy, and surgical reduction, can be considered. Immunomodulatory drugs and monoclonal antibodies have shown success in treating resistant RDD cases. The best approach to treatment remains to be determined and should be personalized based on individual patient factors, including associated diseases and extranodal involvement [89].

The differential diagnosis of the described atypical pelvic tumors in children is presented in Table 1.

## 4. Conclusions

Magnetic resonance imaging is a valuable noninvasive tool in diagnosing both typical and atypical pelvic masses in children. Certain imaging features allow for differential diagnosis between masses and, in the case of typical tumors, a radiological diagnosis consistent with a later histopathologic one is possible. In case of atypical tumors, without additional patient data, such as clinical picture, associated diseases/syndromes, and laboratory test results, a definitive diagnosis cannot be made.

## Figures and Tables

**Table 1:** Differential diagnosis of selected atypical pelvic tumors in children.

Tumor Type	Imaging Features	Abnormalities in Laboratory Tests	Clinical Picture
Small cell carcinoma of the ovary, hypercalcemic type	non-specific; unilateral, sizable mass with central necrosis or calcifications	hypercalcemia	symptoms associated with hypercalcemia: polydipsia, constipation, polyuria, bone pain, muscle weakness
Ewing sarcoma/primitive neuroectodermal tumor of the ovary	variable; three MRI patterns: (1) large, solid, heterogeneous tumor with restriction diffusion and varying degrees of contrast enhancement, (2) mass consisting of thick-walled cystic lesions with numerous septa, (3) combination of (1) and (2)	-	non-specific; abdominal/pelvic pain, mass in the abdomen/pelvis, weight loss, bloating, irregular periods, abnormal vaginal bleeding, back and lower limb pain
Diffuse large B-cell lymphoma of the ovaries	bilateral, homogenous solid masses; “touching” large ovaries, small cysts arranged linearly around the periphery	elevated Ca-125 andLDH levels	non-specific; pain or discomfort in the abdominal cavity, enlarged abdominal circumference, irregular bleeding, B symptoms (fever, night sweats, or weight loss)
Ovarian Sertoli–Leydig cell tumor with rhabdomyosarcoma	variable; solid, solid-cystic, or exclusively cystic; T2 signal depends on amount of fibrous stroma; strong enhancement of solid elements in arterial phase	elevated testosterone level	virilization; associated with DICER1 syndrome
Primary ovarian angiosarcoma	unilateral, sizable, cystic and solid elements in various proportions; hemorrhage; fibrous component	-	non-specific; asymptomatic or neurological symptoms, abdominal pain and distension
Cervical cancer	expansive or infiltrating mass; higher signal intensity than cervical stroma on T2WI, diffusion restriction	-	abnormal vaginal bleeding, vaginal discharge
Neuroblastoma	heterogeneous mass due to calcification, hemorrhage, necrosis; strong diffusion restriction	-	asymptomatic palpable mass; urinary retention
Plexiform neurofibroma	extensive, infiltrative mass spreading along nerve plexuses; high signal on T2WI; “target sign”; “reverse target sign”; no diffusion restriction	-	usually asymptomatic; associated with neurofibromatosis type 1
Rosai–Dorfman disease involving the pelvic and inguinal lymph nodes	nodal form: significantly enlarged pelvic/inguinal lymph nodes; extranodal form: abnormal presacral mass; diffusion restriction; homogeneous contrast enhancement	-	palpable mass in subcutaneous tissue, fever, weight loss, night sweats, bone pain

## Data Availability

No new data were created or analyzed in this study.

## References

[1] Siegel M.J., Hoffer F.A. (2002). Magnetic resonance imaging of nongynecologic pelvic masses in children. Magn. Reson. Imaging Clin. N. Am..

[2] Kwasniewicz P., Wieczorek-Pastusiak J., Romaniuk-Doroszewska A., Bekiesinska-Figatowska M. (2023). Congenital Tumors-Magnetic Resonance Imaging Findings with Focus on Rare Tumors. Cancers.

[3] Behr G.G., Morani A.C., Artunduaga M., Desoky S.M., Epelman M., Friedman J., Lala S.V., Seekins J., Towbin A.J., Back S.J. (2023). Imaging of pediatric ovarian tumors: A COG Diagnostic Imaging Committee/SPR Oncology Committee White Paper. Pediatr. Blood Cancer.

[4] Birbas E., Kanavos T., Gkrozou F., Skentou C., Daniilidis A., Vatopoulou A. (2023). Ovarian Masses in Children and Adolescents: A Review of the Literature with Emphasis on the Diagnostic Approach. Children.

[5] Sahin H., Abdullazade S., Sanci M. (2017). Mature cystic teratoma of the ovary: A cutting edge overview on imaging features. Insights Imaging.

[6] Castle J.T., Levy B.E., Allison D.B., Rodeberg D.A., Rellinger E.J. (2023). Pediatric Rhabdomyosarcomas of the Genitourinary Tract. Cancers.

[7] Ward E., DeSantis C., Robbins A., Kohler B., Jemal A. (2014). Childhood and adolescent cancer statistics, 2014. CA Cancer J. Clin..

[8] Sobieraj P., Malas Z., Issat T., Raciborska A., Bekiesinska-Figatowska M. (2023). Rhabdomyosarcoma of the genitourinary system in girls—The role of magnetic resonance imagining in diagnosis, treatment monitoring, and follow-up. Ginekol. Pol..

[9] Wohlmuth C., Wohlmuth-Wieser I. (2021). Gynecologic Malignancies in Children and Adolescents: How Common is the Uncommon?. J. Clin. Med..

[10] Steliarova-Foucher E., Colombet M., Ries L.A.G., Moreno F., Dolya A., Bray F., Hesseling P., Shin H.Y., Stiller C.A., IICC-3 contributors (2017). International incidence of childhood cancer, 2001–10: A population-based registry study. Lancet Oncol..

[11] Ferguson J.L., Turner S.P. (2018). Bone Cancer: Diagnosis and Treatment Principles. Am. Fam. Physician.

[12] Lima-Bernardes F., Soares D.M., Pereira J.M., Catarino I., Vieira S.E., Carvalho J.D.D. (2021). Pelvic Ewing Sarcoma: The Great Mimicker. Rev. Bras. Ortop..

[13] Atwi D., Quinton M.R., Kiser R.M., Pokala H.R., Rooms L.M., Yu Z. (2021). Small Cell Carcinoma of the Ovary, Hypercalcemic Type, in a 12-Month-Old Girl. Pediatr. Dev. Pathol..

[14] Coşkun Ç., Kurucu N., Usubutun A., Soyer T., Ozcan H.N., Çelik Ertaş N.B., Kutluk T. (2023). Small Cell Carcinoma of Ovary, Hypercalcemic Type: A Rare Case Report. J. Pediatr. Adolesc. Gynecol..

[15] Nasioudis D., Chapman-Davis E., Frey M.K., Caputo T.A., Witkin S.S., Holcomb K. (2018). Small Cell Carcinoma of the Ovary: A Rare Tumor with a Poor Prognosis. Int. J. Gynecol. Cancer.

[16] Wens F.S.P.L., Hulsker C.C.C., Fiocco M., Zsiros J., Smetsers S.E., de Krijger R.R., van der Steeg A.F.W., Zweemer R.P., Baas I.O., Roes E.M. (2023). Small Cell Carcinoma of the Ovary, Hypercalcemic Type (SCCOHT): Patient Characteristics, Treatment, and Outcome-A Systematic Review. Cancers.

[17] Aggarwal D., Gupta P., Chhabra P., Peters N.J., Bansal D., Srinivasan R., Kakkar N. (2021). Small Cell Carcinoma of Ovary, Hypercalcemic Type: Cytologic, Histopathologic, and Immunohistochemical Landscapes of a Rare Case. J. Pediatr. Adolesc. Gynecol..

[18] Kopp L.M., Desoky S., Pugh J., Herzog C.E. (2013). Small cell carcinoma of the ovary of the hypercalcemic type presenting in a 5-year-old girl. J. Pediatr. Hematol. Oncol..

[19] Schleef J., Wagner A., Kleta R., Schaarschmidt K., Dockhorn-Dworniczak B., Willital G., Jürgens H. (1999). Small-cell carcinoma of the ovary of the hypercalcemic type in an 8-year-old girl. Pediatr. Surg. Int..

[20] Sanders B.E., Wolsky R., Doughty E.S., Wells K.L., Ghosh D., Ku L., Pressey J.G., Bitler B.B., Brubaker L.W. (2022). Small cell carcinoma of the ovary hypercalcemic type (SCCOHT): A review and novel case with dual germline SMARCA4 and BRCA2 mutations. Gynecol. Oncol. Rep..

[21] Sholler G.L., Luks F., Mangray S., Meech S.J. (2005). Advanced small cell carcinoma of the ovary in a pediatric patient with long-term survival and review of the literature. J. Pediatr. Hematol. Oncol..

[22] Khosla D., Gupta N., Koshy A., Dalal A., Pandey A.K., Dimri K. (2019). Ovarian Small Cell Carcinoma of Hypercalcemic Type in an Adolescent Girl. J. Obstet. Gynaecol. India.

[23] Murphey M.D., Senchak L.T., Mambalam P.K., Logie C.I., Klassen-Fischer M.K., Kransdorf M.J. (2013). From the radiologic pathology archives: Ewing sarcoma family of tumors: Radiologic-pathologic correlation. Radiographics.

[24] Grigoriu C., Terzea D.C., Lisievici A.C., Georgescu T.A., Constantin A.E., Bacalbaşa N., Ducu I., Bohîlţea R.E. (2021). Peripheral-type primitive neuroectodermal tumor of the ovary with EWSR1-FLI1 fusion transcript: A case report and brief review of literature. Rom. J. Morphol. Embryol..

[25] Hou M.M., Xi M.R., Yang K.X. (2013). A rare case of extraosseous Ewing sarcoma primarily arising in the ovary. Chin. Med. J..

[26] Chao X., Bi Y., Li L. (2019). Ovarian primary primitive neuroectodermal tumor: A review of cases at PUMCH and in the published literature. Orphanet J. Rare Dis..

[27] Nili F., Sedighi Moghadam Pour A., Moradi Tabriz H., Sedighi Moghadam Pour P., Saffar H. (2018). Peripheral Primitive Neuroectodermal Tumor of the Ovary: The Report of Two Rare Cases. Iran. J. Pathol..

[28] Yousefi Z., Sharifhi N., Hasanzadeh M., Mottaghi M., Bolandy S. (2014). Peripheral primitive neuroectodermal tumor of the pelvis. Iran. J. Med. Sci..

[29] Chu L.H., Chang W.C., Kuo K.T., Sheu B.C. (2014). Primary primitive neuroectodermal tumor of the ovary. Taiwan J. Obstet. Gynecol..

[30] Li Y.P., Chang K., Chen T.W., Lee S.P., Chen C.A., Cheng W.F. (2019). Primary Ewing Family of Tumor Arising in the Ovary: A Case Report. Int. J. Gynecol. Pathol..

[31] Sung Y.W., Lin Y.S., Chen Y.T., Yeh L.S. (2022). Non-Hodgkin’s B-cell lymphoma of the ovary: A case report and review of the literature. Taiwan J. Obstet. Gynecol..

[32] Gerrity C., Mercadel A., Alghamdi A., Huang M. (2023). Primary ovarian lymphoma: A case report. Gynecol. Oncol. Rep..

[33] Roik D., Gadomski A., Piotrowski D., Bekiesinska-Figatowska M., Brzewski M. (2011). Burkitt lymphoma with ovary involvement and diffuse peritoneal lymphomatosis in 17-year-old girl with raised CA 125 level—Case report. Nowa Pediatr..

[34] Islimye Taskın M., Gokgozoglu L., Kandemır B. (2013). Primary ovarian large B-cell lymphoma. Case Rep. Obstet. Gynecol..

[35] Kacemi L., Chellaoui M., Dafiri R. (2004). Non-Hodgkin lymphoma of the calcaneus and ovary in an infant. J. Radiol..

[36] Lee A.C., Chui C.H. (2015). Bilateral Ovarian Burkitt’s Lymphoma: Successful Treatment with Preservation of Ovarian Function. J. Pediatr. Adolesc. Gynecol..

[37] Li P.C., Lim P.Q., Hsu Y.H., Ding D.C. (2020). Ovarian Diffuse Large B-cell Lymphoma Initially Suspected Dysgerminoma Managed by Laparoscopic Staging Surgery. Gynecol. Minim. Invasive Ther..

[38] Stepniak A., Czuczwar P., Szkodziak P., Wozniakowska E., Wozniak S., Paszkowski T. (2017). Primary ovarian Burkitt’s lymphoma: A rare oncological problem in gynaecology: A review of literature. Arch. Gynecol. Obstet..

[39] Persano G., Crocoli A., Martucci C., Vinti L., Cassanelli G., Stracuzzi A., Cardoni A., Inserra A. (2023). Case report: Primary ovarian Burkitt’s lymphoma: A puzzling scenario in pediatric population. Front. Pediatr..

[40] Miyazaki N., Kobayashi Y., Nishigaya Y., Momomura M., Matsumoto H., Iwashita M. (2013). Burkitt lymphoma of the ovary: A case report and literature review. J. Obstet. Gynaecol. Res..

[41] Jan Z., Khan A.U., Ilyas A., Faiz S. (2023). Primary Ovarian Burkitts Lymphoma. J. Ayub Med. Coll. Abbottabad.

[42] Zhang Y., Ren M., Hong Y., Zhong Y., Cong X., Chen C., Liu Z., Man Y., Yang L. (2020). Sertoli-Leydig cell tumor in two siblings with DICER1 syndrome: A case report and literature review. Medicine.

[43] Shero N., Dhir A., Bejarano P., Rhode S., Goicocechea J.C. (2024). *DICER1*-related Sertoli-Leydig cell tumor and rhabdomyosarcoma: An evolving disease with a challenging clinical course and treatment: A case report. Case Rep. Womens Health.

[44] Koo J., Garrington T.P., Kerr K., Treece A.L., Cost C.R. (2020). Pediatric ovarian Sertoli-Leydig cell tumors with heterologous rhabdomyosarcoma elements: Clinical case series and review of the literature. Pediatr. Blood Cancer.

[45] Burnik Papler T., Frković Grazio S., Kobal B. (2016). Sertoli—Leydig cell tumor with retiform areas and overgrowth of rhabdomyosarcomatous elements: Case report and literature review. J. Ovarian Res..

[46] Chougule A., Singh P., Saha P.K., Dey P. (2016). Ovarian Sertoli-Leydig cell tumour with rhabdomyosarcoma and borderline mucinous neoplasm. Pathology.

[47] Schultz K.A.P., Stewart D.R., Kamihara J., Bauer A.J., Merideth M.A., Stratton P., Huryn L.A., Harris A.K., Doros L., Field A., Adam M.P., Feldman J., Mirzaa G.M., Pagon R.A., Wallace S.E., Bean L.J.H., Gripp K.W., Amemiya A. (2014). DICER1 Tumor Predisposition. GeneReviews^®^ [Internet].

[48] Stewart C.J., Charles A., Foulkes W.D. (2016). Gynecologic Manifestations of the DICER1 Syndrome. Surg. Pathol. Clin..

[49] Plastini T., Staddon A. (2017). Sertoli-Leydig Cell Tumor with Concurrent Rhabdomyosarcoma: Three Case Reports and a Review of the Literature. Case Rep. Med..

[50] Xu Q., Zou Y., Zhang X.F. (2018). Sertoli-Leydig cell tumors of ovary: A case series. Medicine.

[51] Chen J., Liu Y., Zhang Y., Wang Y., Chen X., Wang Z. (2021). Imaging, clinical, and pathologic findings of Sertoli-leydig cell tumors. Sci. Prog..

[52] Thankamony P., Chandar R., Kattoor J., Nair R.K. (2018). Pediatric Primary Ovarian Angiosarcoma: From Rarity to a Realization. J. Pediatr. Adolesc. Gynecol..

[53] Iljazović E., Tomić S., Mustedanagić-Mujanović J., Karasalihović Z., Kuljanin M., Fatušić Z., Konjić E., Husarić E., Latifagić A., Arnautalić L. (2011). Angiosarcoma of the ovary in an 11 year old girl: Case report and review of the literature. Bosn. J. Basic Med. Sci..

[54] Rehman S., Harikrishna A., Silwal A., Sumie B., Mohamed S., Kolhe N., Maddi M., Huynh L., Gutierrez J., Annepu Y.R. (2024). Ovarian angiosarcoma: A systematic review of literature and survival analysis. Ann. Diagn. Pathol..

[55] Johnson A.M., Argenta P.A. (2023). Angiosarcoma of the ovary treated with polyadenosine ribose polymerase Inhibition, a case report and review of the literature. Gynecol. Oncol. Rep..

[56] Ye H., Lin M., Li R., Qin S., Hou G., Chen H., Li X. (2021). Primary ovarian angiosarcoma: A rare and recognizable ovarian tumor. J. Ovarian Res..

[57] Lee K., Chien T., Huang S., Wang Y.C., Jeng C.M. (2013). Radiological Appearance of Primary Ovarian Angiosarcoma in a 79-Year-Old Woman: A Case Report and Literature Review. J. Radiol. Sci..

[58] Aragon L., Terreros D., Ho H., Greenberg H., Kupesic Plavsic S. (2011). Angiosarcoma of the ovary arising in a mucinous cystadenoma. J. Clin. Ultrasound.

[59] Kudela E., Nachajova M., Biringer K., Slavik P., Plank L., Danko J. (2018). Bilateral ovarian angiosarcoma arising from the mature cystic teratomas—A case report and review of the literature. Int. J. Surg. Case Rep..

[60] Yonezawa I., Waki M., Tamura Y., Onoda R., Narushima M., Ishizuka T., Tajima S. (2014). Gemcitabine-based regimen for primary ovarian angiosarcoma with MYC amplification. Curr. Oncol..

[61] Albertin C., Johnson K.A., Connor J.P., Al-Niaimi A.N. (2013). Angiosarcoma originating from an ovarian mature teratoma, a rare disease with complex treatment modalities. Gynecol. Oncol. Case Rep..

[62] Davidson B., Abeler V.M. (2005). Primary ovarian angiosarcoma presenting as malignant cells in ascites: Case report and review of the literature. Diagn. Cytopathol..

[63] Bujor I.E., Lozneanu L., Ursache A., Cristofor A., Scurtu A.-M., Plamadeala P., Gireada R., Mandici C.E., Găină M.A., Matasariu D.R. (2022). Primary Clear Cell Adenocarcinoma of the Uterine Cervix in a 14-Year-Old Virgin Girl: Case Report. Int. J. Environ. Res. Public Health.

[64] Ferreira R.R., Batista C.S., Millito C.B., Alves G.C., Vieira R.R. (2018). Advanced Uterine Cervix Squamous Cell Carcinoma in 17 Year Old Adolsecent—Case Report. J. Gynecol. Women’s Health.

[65] Evans M., Lawson A., Jarin J.D., Wilson E.E. (2021). Squamous Carcinoma of the Cervix in a 15-Year-Old with Congenital HIV: A Case Report. J. Pediatr. Adolesc. Gynecol..

[66] Lawson A.A., Wilson E. (2019). Cervical cancer in Adolescent with HIV: A Case Report. J. Pediatr. Adolesc. Gynecol..

[67] Re G.L., Cucinella G., Zaccaria G., Crapanzano A., Salerno S., Pinto A., Casto A.L., Chiantera V. (2023). Role of MRI in the Assessment of Cervical Cancer. Seminars in Ultrasound, CT and MRI.

[68] Mansoori B., Khatri G., Rivera-Colon G., Albuquerque K., Lea J., Pinho D.F. (2020). Multimodality imaging of uterine cervical malignancies. Am. J. Roentgenol..

[69] Swift C.C., Eklund M.J., Kraveka J.M., Alazraki A.L. (2018). Updates in Diagnosis, Management, and Treatment of Neuroblastoma. Radiographics.

[70] Nour-Eldin N.E., Abdelmonem O., Tawfik A.M., Naguib N.N., Klingebiel T., Rolle U., Schwabe D., Harth M., Eltoukhy M.M., Vogl T.J. (2012). Pediatric primary and metastatic neuroblastoma: MRI findings: Pictorial review. Magn. Reson. Imaging.

[71] Shields L.B.E., Peppas D.S., Rosenberg E. (2019). Pelvic neuroblastoma presenting with acute urinary retention and acute kidney injury. Urol. Case Rep..

[72] Littooij A.S., de Keizer B. (2023). Imaging in neuroblastoma. Pediatr. Radiol..

[73] Rasmussen A., Muñiz A.E., King B. (2010). Neuroblastoma causing acute urinary retention: A rare presentation. J. Emerg. Med..

[74] Chen A.M., Trout A.T., Towbin A.J. (2018). A review of neuroblastoma image-defined risk factors on magnetic resonance imaging. Pediatr. Radiol..

[75] Goo H.W. (2010). Whole-body MRI of neuroblastoma. Eur. J. Radiol..

[76] Gahr N., Darge K., Hahn G., Kreher B.W., von Buiren M., Uhl M. (2011). Diffusion-weighted MRI for differentiation of neuroblastoma and ganglioneuroblastoma/ganglioneuroma. Eur. J. Radiol..

[77] Belakhoua S.M., Rodriguez F.J. (2021). Diagnostic Pathology of Tumors of Peripheral Nerve. Neurosurgery.

[78] Messersmith L., Krauland K. (2024). Neurofibroma. StatPearls [Internet].

[79] Zulfiqar M., Lin M., Ratkowski K., Gagnon M.H., Menias C., Siegel C.L. (2021). Imaging Features of Neurofibromatosis Type 1 in the Abdomen and Pelvis. AJR Am. J. Roentgenol..

[80] Zacharia T.T., Jaramillo D., Poussaint T.Y., Korf B. (2005). MR imaging of abdominopelvic involvement in neurofibromatosis type 1: A review of 43 patients. Pediatr. Radiol..

[81] Jana M., Gamanagatti S., Kumar R., Aggarwala S. (2011). Pelvic neurofibroma arising from prostate in a case of neurofibromatosis-1. Indian J. Urol..

[82] Miraglia E., Laghi A., Moramarco A., Giustini S. (2022). Juvenile xanthogranuloma in neurofibromatosis type 1. Prevalence and possible correlation with lymphoproliferative diseases: Experience of a single center and review of the literature. Clin. Ter..

[83] Ferrari F., Masurel A., Olivier-Faivre L., Vabres P. (2014). Juvenile xanthogranuloma and nevus anemicus in the diagnosis of neurofibromatosis type 1. JAMA Dermatol..

[84] Ozcan H.N., Karcaaltincaba M., Oguz B., Haliloglu M. (2014). Radiological manifestations of abdominopelvic nerve tumours seen in neurofibromatosis type 1. Clin. Radiol..

[85] Grover D.S.B., Kundra D.R., Grover D.H., Gupta D.V., Gupta D.R. (2021). Imaging diagnosis of plexiform neurofibroma- unravelling the confounding features: A report of two cases. Radiol. Case Rep..

[86] Well L., Salamon J., Kaul M.G., Farschtschi S., Herrmann J., Geier K.I., Hagel C., Bockhorn M., Bannas P., Adam G. (2019). Differentiation of peripheral nerve sheath tumors in patients with neurofibromatosis type 1 using diffusion-weighted magnetic resonance imaging. Neuro Oncol..

[87] Tortora S., Esposito A., Della Pepa G., Paternò M., Cagnoli G.A., Cesaretti C., Natacci F., Carrafiello G. (2021). Neurofibromatosis Type 1 with Neck and Thoraco-Abdominal Involvement: A Case Series Showing Different Localization and MRI Features. Rep. Med. Imaging.

[88] Karajgikar J., Grimaldi G., Friedman B., Hines J. (2016). Abdominal and pelvic manifestations of Rosai-Dorfman disease: A review of four cases. Clin. Imaging.

[89] Hartmann T., Solomon N., Lerner G., Ehrlich L. (2023). Rosai-Dorfman Disease in a Pediatric Patient: Imaging Findings and Pathology with a brief review of the Literature. J. Radiol. Case Rep..

[90] Mar W.A., Yu J.H., Knuttinen M.G., Horowitz J.M., David O., Wilbur A., Menias C.O. (2017). Rosai-Dorfman Disease: Manifestations Outside of the Head and Neck. AJR Am. J. Roentgenol..

